# Why is endoscopic reintervention easier using a braided metal stent than a laser-cut stent in stent-in-stent placement? A bench study

**DOI:** 10.1055/a-2471-8065

**Published:** 2024-12-03

**Authors:** Mitsuru Okuno, Fumiya Kataoka, Atsushi Tagami, Hiroshi Araki, Eiichi Tomita, Hisataka Moriwaki, Masahito Shimizu

**Affiliations:** 1 73505 Gastroenterology, Matsunami General Hospital, Hashima-gun, Japan; 2 476117 First Department of Internal Medicine, Gifu University Hospital, Gifu, Japan

Bilateral stent placement results in better clinical palliation than unilateral placement for unresectable malignant hilar biliary obstruction 1 . Moreover, for simultaneous bilateral drainage, the stent-in-stent method with self-expandable metal stents (SEMSs) shows longer stent patency than side-by-side-placement 1 2 ; however, endoscopic reintervention in stent-in-stent cases is challenging because the additional drainage device must pass through the crossed-wire wall of the inner SEMS ( Fig. 1 ). In such conditions, endoscopic reintervention is more difficult in cases where a laser-cut SEMS has been placed than in those where a braided SEMS was placed 3 , although the reason for this difference has not been satisfactorily explained. We conducted a bench study to address this question, using a video that directly recorded the path of the inserted drainage device, a plastic stent, through the crossed-wire wall of two SEMSs ( Video 1 ).

**Fig. 1:**
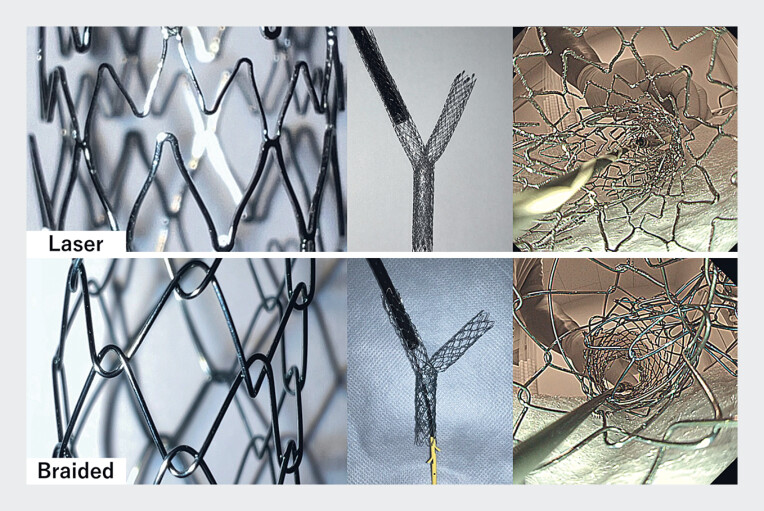
Evaluation of the paths of plastic stents being inserted into stent-in-stent laser-cut or braided self-expandable metal stents (SEMSs) showing a guidewire that was positioned through a slim endoscope, inserted into the 10-mm stent-in-stent laser-cut or braided SEMSs from the intrahepatic bile duct side, then a 7-Fr plastic stent that is inserted from the other side toward the endoscope.

Bench test to investigate the reason that endoscopic reintervention is more difficult in cases with stent-in-stent laser-cut self-expandable metal stents (SEMSs) showing easy insertion of a plastic stent into a braided SEMS but blocked plastic stent progression through a laser-cut SEMS owing to the difference in their structures.Video 1

**Laser-cut SEMS** The view from the intrahepatic bile duct (IHBD) shows a “W”-shaped wire in the center of the hole. This W-shaped wire is located “independently” in the center, as well as on the periphery when the SEMS is folded ( Fig. 2 **a–d** ), thereby making the path for the plastic stent narrower. The plastic stent being inserted is blocked by the W-shaped wire, and therefore the laser-cut SEMS makes plastic stent insertion difficult. In an example from clinical practice, the laser-cut wire catches the endoscopic nasobiliary drainage tube, which cannot be further inserted into the IHBD ( Fig. 2 **e** ).

**Fig. 2:**
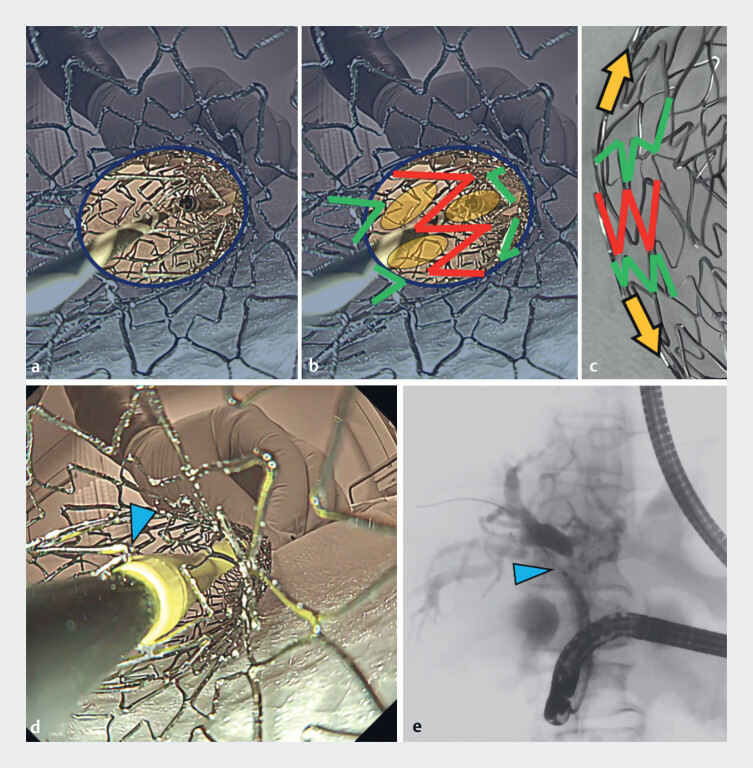
The inside view of the laser-cut self-expandable metal stent (SEMS) from the intrahepatic bile duct side showing: **a** the “W”-shaped wire that is located in the center of the hole; **b, c** how the W-shaped wire is “independently” located in the center and on the periphery, when the SEMS is folded owing to the structure of the laser-cut SEMS; **d** the inserted plastic stent that is met and blocked by the W-shaped wire (arrowhead); **e** radiographic image from clinical practice showing the laser-cut wire catching the endoscopic nasobiliary drainage tube, which cannot proceed further into the intrahepatic bile duct (arrowhead).

**Braided SEMS** Although the wire is also located in the center of the hole, the space for passage of the plastic stent is larger and allows the plastic stent to be passed through easily. The braided SEMS has a “hook-and-cross” structure, in which all wires are linked at the crossing points. Therefore, when a braided SEMS is folded, the wires are pulled from all directions, resulting in the creation of a larger space ( Fig. 3 **a–d** ). In an example from clinical practice, a plastic stent can easily be passed through the crossed wires of a braided SEMS ( Fig. 3 **e** ).

**Fig. 3:**
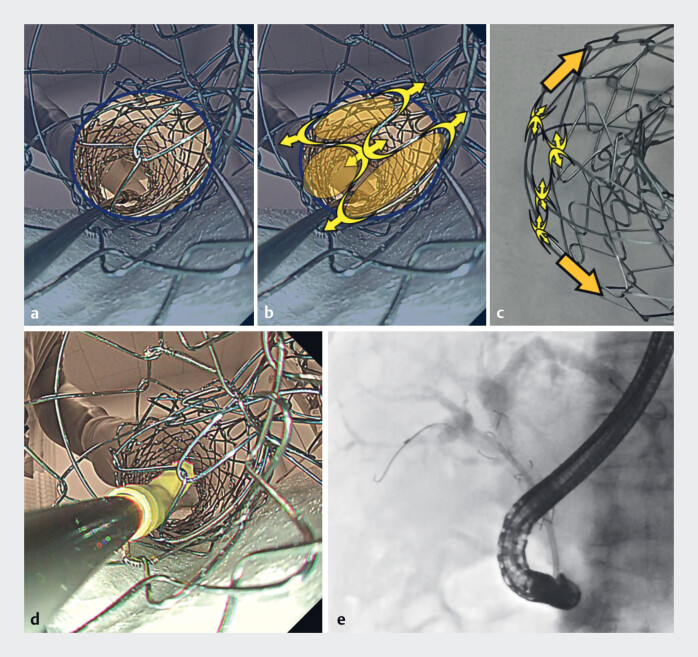
The inside view of a braided self-expandable metal stent (SEMS) from the intrahepatic bile duct side showing: **a** the wire located in the center of the hole, which offers a larger space for passage of the plastic stent than with the laser-cut SEMS; **b, c** how the “hook-and-cross” structure of the braided SEMS, in which all wires are linked at the wire crossing points, pulls the wires from all directions; **d** the larger space that is created for passage of the plastic stent when the braided SEMS is folded; **e** radiographic image from clinical practice showing that a plastic stent can easily be passed through the crossed wires of a braided SEMS.

Braided SEMSs appear to offer easier insertion of a plastic stent for stent-in-stent-placed cases owing to the difference in the structure of the SEMSs. Appropriate SEMS selection is helpful in ensuring safe clinical practice for endoscopic reintervention.

Endoscopy_UCTN_Code_TTT_1AR_2AZ

## Notes

Endoscopy E-Videos https://eref.thieme.de/e-videos
*E-Videos* is an open access online section of the journal *Endoscopy* , reporting on interesting cases and new techniques in gastroenterological endoscopy. All papers include a high-quality video and are published with a Creative Commons CC-BY license. Endoscopy E-Videos qualify for HINARI discounts and waivers and eligibility is automatically checked during the submission process. We grant 100% waivers to articles whose corresponding authors are based in Group A countries and 50% waivers to those who are based in Group B countries as classified by Research4Life (see: https://www.research4life.org/access/eligibility/ ). This section has its own submission website at https://mc.manuscriptcentral.com/e-videos .
